# Dual pathology as a cause of proteinuria in the post-transplant period; report of a case

**Published:** 2015-12-21

**Authors:** Rohit Tewari, Satish Mendonca, Vijay Nijhawan

**Affiliations:** ^1^Department of Pathology, Armed Forces Medical College Pune, India; ^2^Classified Specialist Medicine and Nephrology, Base Hospital Delhi Cantt, India

**Keywords:** Proteinuria, Post-transplant, Recurrent glomerulonephritis, Transplant glomerulopathy

## Abstract

Proteinuria is common after renal transplantation and affects between 35%-45% of patients during the same year as their transplant. We report a case of dual pathology in the renal allograft as a cause of severe proteinuria. A 38-year-old male presented with end-stage renal disease. He underwent live related renal allograft transplant. His immediate post-transplant period was unremarkable. He developed rise in serum creatinine (2.1 mg/dl) 6 months after transplant and was biopsied. He was diagnosed as a case of acute cellular rejection type Ib with suspicion for antibody mediated rejection. He was treated with methylprednisolone to which he showed a good response with return of serum creatinine to 1.6 mg/dl. Subsequently, he developed a nephrotic range proteinuria 6 months after this episode of rejection. Repeat biopsy was performed. He was diagnosed as a case of immune complex mediated glomerulonephritis (GN) (morphologically consistent with pattern of membranoproliferative glomerulonephritis) with chronic humoral rejection in the form of transplant glomerulopathy (TG). IHC for C4d and immunofluorescence studies were instrumental making the diagnosis. He was treated with steroids and rituximab to which he showed a good response with remission of proteinuria. This case highlights the importance of picking up dual pathology in an allograft biopsy to ensure appropriate therapy. The role of C4d and its correct interpretation is further highlighted, especially with regard to pattern (granular versus linear) and location (glomerular capillaries versus peritubular capillaries).

**Implication for health policy/practice/research/medical education:**

This case brings out the importance of thorough evaluation of proteinuria in the post-transplant period with an allograft biopsy due to its varied etiology. The role of C4d immunohistochemistry is further highlighted in distinguishing transplant glomerulopathy (TG) and recurrent glomerulonephritis.

## Introduction

Graft dysfunction in the post-transplant period is a cause of severe concern not only to the treating physician, but also to the patient. The etiology is varied and may include various forms of rejection, recurrence of basic disease, development of fresh kidney disease, calcineurin inhibitor toxicity and development of infections secondary to immunosuppression. Recurrence of glomerulonephritis (GN) and the occurrence of new GN (*de novo* GN) in the transplanted kidney have been reported since the early days of transplantation (1). The advances made in the use of immunosuppressives have greatly influenced the outcome of the cases of rejection. There have been improvements in short- and long-term graft survival after kidney transplantation in the past 2 decades. It is estimated that approximately 10% to 20% of patients with GN develop recurrence in the allograft and 50% of then lose their graft on long-term follow-up. Thus having a negative influence on long-term graft survival. Coexistence of a chronic rejection with this disease may further influence the outcome.

Proteinuria is common after renal transplantation and affects between 35%-45% of patients during the same year as their transplant (2). Post-transplant nephrotic syndrome has distinctive clinicopathologic features with pathogenetic and therapeutic implications (3). This case highlights the importance of detailed evaluation of post-transplant proteinuria in order to arrive at correct etiological diagnosis.

## Case presentation

**Figure 1:**
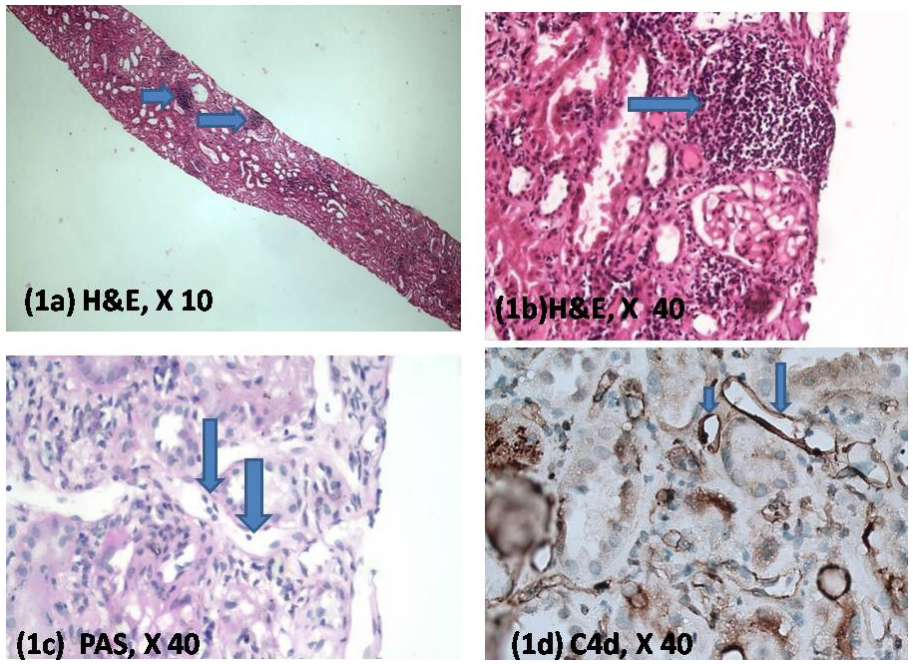
(a) Section through kidney biopsy showing patchy lymphomononuclear infiltrates at scanner magnification (horizontal arrows) (H&E X 10). (b) Higher power view showing details of the same infiltrate (horizontal arrow) (H&E X 40). (c) Section showing dilated peritubular capillaries containing lymphomononuclear cells (vertical arrows) (PAS, X 40). (d) Immunohistochemistry for C4d showing strong linear staining along the peritubular capillaries (vertical arrow).

A 32-year-old male, presented with end-stage renal disease in 2009. The basic disease was unknown. He underwent live related renal allograft transplant with mother as donor and a haplo-match on HLA typing. He was placed on triple immunosuppression with tacrolimus, mycophenolate mofetil and steroids. His immediate post-transplant period was unremarkable. He developed rise in serum creatinine (2.1 mg/dl), 6 months after transplant and was biopsied (Figure 1). Biopsy was adequate and showed a moderate degree of interstitial inflammation with tubulitis. However, there was no vasculitis. Peritubular capillary dilatation and margination were present. Immunohistochemistry for C4d was positive in the peritubular capillaries. He was diagnosed as a case of acute cellular rejection type Ib with suspicion for antibody mediated rejection and donor specific antibody studies were advised. Patient was treated with methylprednisolone to which he showed a good response with return of serum creatinine to 1.6 mg/dl.

**Figure 2:**
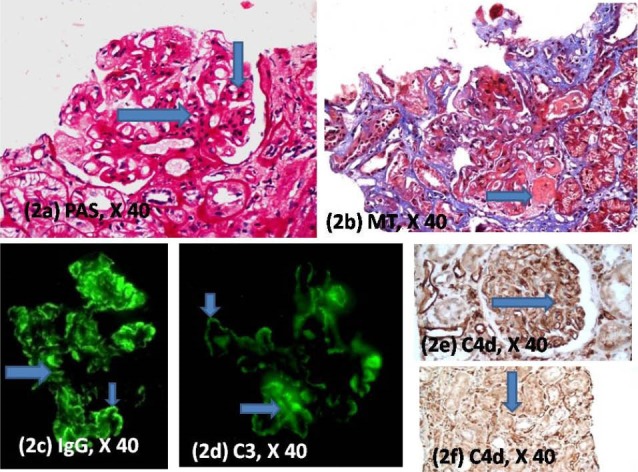
(a) Section through kidney biopsy showing enlarged glomerulus with lobular accentuation and mesangial (horizontal arrow)and segmental endocapillary (vertical arrow) proliferation (PAS X 40). (b) Another glomerulus with similar features showing fuchsinophillic immune complex deposits (horizontal arrow) (Masson Trichrome X 40). (c) Immunofluorecence for IgG showing 3+ granular positivity in the mesangium (horizontal arrow) and the glomerular capillary walls (vertical arrow) (IgG X 40). (d) Immunofluorescence for C3 showing 3+ granular positivity in the mesangium (horizontal arrow) and the glomerular capillary walls (vertical arrow) (C3 X 40). (e) Immunohistochemistry for C4d showing strong granular positivity in the glomerular capillaries (horizontal arrow) (C4D x 40). (f) Immunohistochemistry for C4d showing strong linear positivity in the peritubular capillaries (vertical arrow) (C4d X 40).

Subsequently, patient developed a nephrotic range proteinuria six months after this episode of rejection. Serum creatinine now had risen to 2.8 mg/dl. A repeat biopsy was performed (Figure 2). The new biopsy showed six glomeruli, all of which showed enlargement, accentuated lobulation and increased cellularity with mesangial and endocapillary proliferation. There were not necrotizing lesions or crescents. Periodic acid-Schiff (PAS) and silver stains showed splitting of the glomerular capillary walls. Immunofluorescence studies showed 3+ diffuse granular positivity for IgG and C3 in the mesangium and in the glomerular capillary walls. There was a 1+ positivity for C1q was also noted in the same locations. Immunohistochemistry for C4d was performed which showed granular positivity in the glomerular and linear positivity in the peritubular capillaries. He was diagnosed as a case of immune-complex mediated GN (morphologically consistent with pattern of membranoproliferative GN) with chronic humoral rejection in the form of transplant glomerulopathy (TG). His immunosuppression was increased to oral prednisolone (1 mg/Kg). He was also given two injection doses of Rituximab 375 mg/m^2^, 2 weeks apart. Evaluation subsequently revealed a stable renal function with a serum creatinine of 2 mg/dl and is in remission for proteinuria. His steroids have been tapered to a maintenance dose of 7.5 mg/day along with tacrolimus and mycophenolate mofetil.

## Discussion

The presence of proteinuria in the post-transplant period needs to be carefully evaluated. The first consideration is whether the proteinuria is coming from the allograft or from the native kidneys. Compared with patients without proteinuria, those with proteinuria have a higher incidence of glomerular diseases in the allograft (4). A study conducted by Myslak et al (5) brought out certain interesting conclusions. Post-transplant proteinuria of >3 grams/day could not be attributed to the native kidneys in patients with well functioning grafts, even if the pre-transplant level of proteinuria was nephrotic range. Second, proteinuria greater than 1500 mg/day, one year post-transplant and/or an increase in proteinuria from 3 weeks to one year >500 mg/d is suggestive of new allograft pathology. Third, native kidneys may be responsible for low levels of proteinuria (<500 mg/day) in transplant recipients even one year after transplantation. However, native proteinuria is expected to decline over time after transplantation (5). In our case, the proteinuria was significantly high and could be ascribed to graft pathology.

Our case quite clearly had an immune complex mediated GN evidenced by the characteristic glomerular morphology along with supportive immunofluorescence studies. However, he also had history of an episode of rejection earlier (both acute cellular and antibody mediated rejection). Considering this, presence of associated rejection could not be discounted and a stain for C4d was mandatory. The thickening and splitting of the glomerular capillary walls along with C4d positivity helped in making a diagnosis of associated TG.

Originally classified as a variant of chronic allograft nephropathy of unknown etiology, TG is now known to occur in cases with a past history of antibody mediated graft rejection, and it is associated with the deposition of the complement degradation product C4d, which suggests that TG may be one manifestation of antibody-mediated graft injury (6,7). The interpretation of the stain for C4d in this case was critical to making the correct diagnosis. The stain for C4d revealed linear positivity in the peritubular capillaries (indicating the presence of a chronic humoral rejection) and granular positivity in the glomerular capillaries (supporting the presence  of an immune complex mediated GN (8). The presence of a previous episode of acute antibody-mediated rejection further supports the development of TG. The role of electron microscopy in this case is important. However, we did not have access to the facility. Electron microscopy would have been helpful in picking up immune complex mediated GN. However the presence of positive immunofluorescence for IgG and C3 and availability of C4d immunostaining helped in clarifying the situation. The role of C4d in a proliferative GN was explored by Sethi et al recently (8). They inferred that staining pattern of C4d mirrored the staining patterns of IgG and C3 in immune complex mediated GN. The staining pattern was also described as being granular, as against the linear pattern characteristic of antibody mediated rejection.

A comment on whether the proliferative GN was recurrent or de novo was not possible, since the basic disease in this case was unknown. However, the occurrence of a proliferative GN along with a TG does explain the severe proteinuria that this patient had presented with.

## Conclusion

This case highlights the importance of picking up dual pathology in an allograft biopsy to ensure appropriate therapy. The role of C4d and its correct interpretation is further highlighted, especially with regard to pattern (granular versus linear) and location (glomerular capillaries versus peritubular capillaries).

## Authors’ contribution

RT; case description, work up and drafting of manuscript. SM; management of case and manuscript preparation. VN; supervision

## Conflicts of interest

The author declared no competing interests.

## Ethical considerations

Ethical issues (including plagiarism, data fabrication, double publication) have been completely observed by the authors.

## Funding/Support

None.

## References

[1] Hariharan S, Peddi VR, Savin VJ, Johnson CP, First MR, Roza AM, Adams MB (1998). Recurrent and de novo renal diseases after renal transplantation: A report from the renal allograft disease registry. Am J Kidney Dis.

[2] Knoll GA (2009). Proteinuria in kidney transplant recipients: Prevalence, prognosis, and evidence-based management. Am J Kidney Dis.

[3] Yakupoglu U, Baranowska-Daca E, Rosen D, Barrios R, Suki WN, Truong LD (2004). Post-transplant nephrotic syndrome: A comprehensive clinicopathologic study. Kidney Int.

[4] Amer H, Cosio FG (2009). Significance and management of proteinuria in kidney transplant recipients. J Am Soc Nephrol.

[5] Myslak M, Amer H, Morales P, Fidler ME, Gloor JM, Larson TS (2006). Interpreting post-transplant proteinuria in patients with proteinuria pre-transplant. Am J Transplant.

[6] Racusen LC, Solez K, Colvin RB, Bonsib SM, Castro MC, Cavallo T (1999). The banff 97 working classification of renal allograft pathology. Kidney Int.

[7] Nankivell BJ, Alexander SI (2010). Rejection of the kidney allograft. N Engl J Med.

[8] Sethi S, Nasr SH, De Vriese AS, Fervenza FC (2015). C4d as a diagnostic tool in proliferative GN. J Am Soc Nephrol.

